# Supplementary material for: An LLM chatbot to facilitate primary-to-specialist care transitions: a randomized controlled trial
Source: Nat Med. 2026 Jan 19;32(3):934–42. doi: 10.1038/s41591-025-04176-7 (PMC13004692; doi:10.1038/s41591-025-04176-7)
Supplement: Supplementary file 1 — Supplementary Tables 1–8. Study protocol and statistical analysis plan (English). Study protocol and statistical analysis plan (Chinese). [file 41591_2025_4176_MOESM1_ESM.pdf]

nature medicine

Supplementary information https://doi.org/10.1038/s41591-025-04176-7

An LLM chatbot to facilitate primary-to-specialist care transitions: a randomized controlled trial

In the format provided by the authors and unedited

Supplementary Tables and Supplementary Figures

Table of Contents

Supplementary Table 1 The survey questionnaires for participating patients and caregivers 2

Supplementary Table 2 The survey questionnaires for physicians 4

Supplementary Table 3 Contrast of dialogues between the codesigned model and a simulated patient with its local data-tuned counterpart and the same simulated patient... 6

Supplementary Table 4 Three examples of referral reports generated by PreA 11

Supplementary Table 5 The referral report evaluation rubrics 17

Supplementary Table 6 A de-identified case of PreA interacting with real-world patients: The patient missed important information until later in the dialogue that required multi-disciplinary assessment 19

Supplementary Table 7 A de-identified case of PreA interacting with real-world patients: The patient took medications without consulting a doctor 22

Supplementary Table 8 A de-identified case of PreA interacting with real-world patients: The patient had concerns about examinations 25

Supplementary Table 1 The survey questionnaires for participating patients and caregivers

Citeria	Questions	Single Chocies
Easy of communication	During your conversation, how easy was it to have a conversation with the doctor?	Very difficult, I completely unsure how to converse with the doctor
		Difficult, the doctor often asked questions that were hard to understand, and I didn't know how to respond
		Neutral
		Pretty easy, our conversation was smooth
		Very easy, our conversation was relaxed and comfortable
Physician attentiveness	During the conversation, did you feel the doctor spent enough time focusing on you?	Very little
		Quite little
		Okay
		Quite a lot
		Plenty
Interpersonal regard	Did you feel friendly and respected during this consultation service?	Not at all friendly, the doctor only asked questions mechanically, ignored my feedback
		Often unfriendly, the doctor did not explain my doubts
		Okay, the doctor may proactively explain complex medical terms to me
		Mostly friendly, the doctor could explain most of my doubts

		Very friendly, the doctor could patiently explain all my doubts
Patient satisfaction	Overall, how satisfied were you with the consultation service?	Very dissatisfied
		Dissatisfied
		Okay
		Quite satisfied
		Very satisfied
Future acceptability	If you get sick or worried about your health next time, would you be willing to use AI health consultation to get a preliminary understanding of your health problem?	Definitely not, there's no need
		Not really, I'm not very familiar with using it yet
		Uncertain, it's hard to say
		Yes, so I can communicate better with the doctor
		Definitely, so I can communicate better with the doctor and even avoid unnecessary visits to the hospital

Notes: The initial Chinese questionnaires have been translated into English and carefully reviewed to maintain their original meaning. Specifically, the principal investigator (SH) translated the Mandarin text into English. Two researchers (XT and SZ) compared the English and Chinese versions line by line to resolve semantic and cultural discrepancies.

Supplementary Table 2 The survey questionnaires for physicians

Citeria	Questions	Single Chocies
Usefulness in decision-making	How useful was PreA assistant in your clinical decision-making?	Not useful at all
		Not useful
		Hard to say
		Useful
		Very useful
	[If the answer is "Very useful" or " Useful", please select all that apply:] What did you find most useful or impressive? (Multiple choice)	Interpreting diagnostic reports to patients
		Efficiently inquiring and recording medical history
		Assisting cross-disciplinaries medical consultation
		Providing preliminary diagnosis suggestions
		Helping me learn how to better communicate with patients
Relief of workload	Does PreA assistant reduce your workload?	Definitely not
		Not realy
		Uncertain
		Yes

		Definitely yes
Ease of communication	Do you believe a PreA assistant would improve communication with patients?	Definitely not
		Not really
		Uncertain
		Yes
		Definitely yes

Notes: The initial Chinese questionnaires have been translated into English and carefully reviewed to maintain their original meaning. Specifically, the principal investigator (SH) translated the Mandarin text into English. Two researchers (XT and SZ) compared the English and Chinese versions line by line to resolve semantic and cultural discrepancies.

Supplementary Table 3 Contrast of dialogues between the codesigned model and a simulated patient with its local data-tuned counterpart and the same simulated patient

Co-designed model	Data-tuned counterpart	Comparison
**Patient:**<br>Doctor, I've been feeling off lately—my weight dropped suddenly, and there are some other issues too.<br><br>**Doctor:**<br>Thank you for sharing. [3] First, I'd like more details. How old are you, and what is your gender?[1]<br><br>**Patient:**<br>I'm 28, female.<br><br>**Doctor:**<br>Thank you. You mentioned sudden weight loss. Could you share specifics? For example, how many kilograms have you lost, and over how long? [1]<br><br>**Patient:**<br>Over the past few months, I've lost around 5–6 kilograms. I now weigh 60 kilograms.<br><br>**Doctor:**<br>Understood. Losing 5–6 kilograms in months is notable. Beyond weight loss, do you have other	**Patient:**<br>Doctor, recently I just haven't been feeling quite right. My weight has dropped inexplicably. Plus, my mouth is always sore. These symptoms are making me quite worried.<br><br>**Doctor:**<br>I understand all of these issues you've mentioned. I'll ask you two or three more questions. Have you been constipated recently? [1]<br><br>**Patient:**<br>Um, doctor, regarding my bowel movements, I haven't felt particularly constipated recently, they've been normal.<br><br>**Doctor:**<br>Do you often feel thirsty at night and want to drink water? [1]<br><br>**Patient:**<br>No, Doctor. I don't really feel thirsty at night. I don't have much desire to drink water in general.	**[1] Doctor's detailed inquiry**<br>- Guides systematic data collection (e.g., age and sex).<br>- Asks specific follow-ups (duration, associated symptoms).<br><br>**[2] Diagnositci logic**<br>- Provides differential diagnosis.<br>- Integrates family history and risk factors.<br>- Evidence-based testing recommendations.<br><br>**[3] Tone and empathy**<br>- Uses empathetic language ("warrant attention") to build trust.<br><br>**[4] Actionable advice**<br>- Specifies tests (e.g., CT/MRI).<br>- Emphasizes follow-up steps to improve patient compliance.

symptoms—changes in appetite, fatigue, mood swings, or sleep issues?<br><br>**Patient:**<br>Well, I’ve had severe bad breath, occasional difficulty swallowing, loose teeth, persistent oral pain, and a lump on my neck. All these feel very concerning.<br><br>**Doctor:**<br>Thank you for detailing these symptoms. They do require attention. [3] To assist further: Have you experienced pain, fever, major illnesses, or chronic conditions recently? Any diagnosed medical history or medications? [1]<br><br>**Patient:**<br>No medications or chronic conditions. I was healthy until these months. My mother has diabetes, and my uncle was diagnosed with oral cancer—that’s our family history.<br><br>**Doctor:**<br>Thank you for the thorough details and family history. [3] Based on your symptoms (weight loss, bad breath, dysphagia, oral pain, neck lump) , I’d consider the following possible diagnosis and tests:<br><br>**Possible diagnoses directions**[2]	**Doctor:**<br>Your case is quite complex. [3] One last question: Does your mouth feel dry during meals, or do you still have saliva?<br><br>**Patient:**<br>Doctor, during meals, I do still have saliva. It doesn’t feel overly dry.<br><br>**Doctor:**<br>Since you still have saliva, maintain an appetite, and don’t feel overly fatigued, let’s analyze these symptoms. They don’t seem to align with diabetes, do they? [2] What’s your own take?<br><br>**Patient:**<br>Well, Doctor, I’m not really knowledgeable about this. I just noticed these unusual changes in my body and wanted your expertise. Thank you.<br><br>**Doctor:**<br>As I said, you still produce saliva. Review these symptoms again—they don’t point to diabetes. [3]<br><br>**Patient:**<br>Thank you, Doctor. I truly can’t figure this out myself. I just hope you can help identify the issue.	

1. **Thyroid Disorder (e.g., Hyperthyroidism)**<br>o *Supporting Evidence:*<br>▪ Weight loss<br>o *Contradictory Evidence:*<br>▪ Oral symptoms less commonly linked<br>2. **Oral Cancer**<br>o *Supporting Evidence:*<br>▪ Family history (uncle's oral cancer)<br>▪ Oral pain, dysphagia, neck lump<br>o *Contradictory Evidence:*<br>▪ No mentioned risk factors (e.g., smoking, alcohol)<br>3. **Infectious Disease (e.g., oral infection, lymphadenopathy)**<br>o *Supporting Evidence:*<br>▪ Oral pain, neck lump<br>o *Contradictory Evidence:*<br>▪ Requires further symptom confirmation<br><br>**Recommended Tests** [4]<br>1. **Thyroid Function Tests**<br>o *Rationale:* Assess weight loss and potential thyroid issues.<br>2. **Oral Exam & Imaging (e.g., CT/MRI)**<br>o *Rationale:* Investigate oral pain and neck lump.	**Doctor:**<br>Has anyone in your family had diabetes?<br><br>**Patient:**<br>Yes, Doctor. My mother has diabetes, and my uncle was diagnosed with oral cancer.<br><br>**Doctor:**<br>Listen carefully: These symptoms still don't relate to diabetes or your family history. [3] They're unrelated to those conditions.<br><br>**Patient:**<br>Thank you, Doctor. What do you think might be causing these symptoms? I just want clarity.<br><br>**Doctor:**<br>It likely has little to do with your family history.<br><br>**Patient:**<br>Thank you. Could these symptoms link to other health issues? Please advise.<br><br>**Doctor:**<br>Given your case, you must visit an ENT specialist. [2] If symptoms persist as they are, medication might temporarily fixes it—but tumors or chronic conditions at your age require proper evaluation. [4]	

3. **Blood Tests**<br>○ *Rationale:* Screen for infection, inflammation, or systemic issues.<br><br>**Doctor:**<br>Below is a summary of this consulation for you:<br>• **Patient Profile:**<br>○ 28-year-old female<br>○ Chief Complaints: 5–6 kg weight loss, halitosis, dysphagia, oral pain, neck lump.<br>• **History:**<br>○ Recent onset, no medications, family history of diabetes and oral cancer.<br>• **Next Steps:** [4]<br>○ Prioritize thyroid/oral exams and bloodwork.<br><br>**Doctor:**<br>How do you feel about proceeding with these tests?[4]<br><br>**Patient:**<br>Thank you, Doctor. I'll schedule these tests immediately.<br>**Doctor:**<br>You're welcome! Please act promptly and update your doctor. [4] Reach out anytime if	**Patient:**<br>Thank you, Doctor.<br><br>**Doctor:**<br>Take care.<br><br>**Patient:**<br>Understood. Thank you, Doctor. Goodbye.	

questions arise. Wishing you a speedy recovery!<br><br>**Patient:**<br>No further questions. Thank you!		

Notes: The initial Chinese questionnaires have been translated into English and carefully reviewed to maintain their original meaning. Specifically, the principal investigator (SH) translated the Mandarin text into English. Two researchers (XT and SZ) compared the English and Chinese versions line by line to resolve semantic and cultural discrepancies.

Supplementary Table 4 Three examples of referral reports generated by PreA

Example 1:

Patient Demographics

- **Sex**: Female
- **Age**: 46 years
- **Allergy History**: Unknown

Clinical Summary

- **Referred Departments**: Gastroenterology/Oncology
- **Chief Complaint**: Frequent bowel movements (7–8 times daily) accompanied by nausea and vomiting, persisting for 20 days.
- **History of Present Illness**: The patient underwent postoperative chemotherapy for rectal cancer, after which she developed frequent bowel movements and intermittent vomiting with nausea. Symptoms have persisted for 20 days without significant abdominal pain, hematochezia, or fever.
- **Past Medical History**: Rectal cancer surgery.

Symptomatology

- **Positive Symptoms**:
  - Frequent bowel movements (7–8 times daily)
  - Nausea
  - Occasional vomiting
- **Negative Symptoms**:
  - No significant abdominal pain
  - No marked loss of appetite
  - No hematochezia
  - No signs of systemic infection (e.g., fever)

Proposed Diagnostic Workup

1. **Stool routine examination**: To assess for intestinal infection or inflammation.
2. **Colonoscopy**: To evaluate intestinal integrity and rule out secondary pathologies.

3. **Electrolyte panel**: To monitor electrolyte imbalances secondary to vomiting and diarrhea.

Differential Diagnoses

1. **Chemotherapy-induced acute intestinal reaction**
   - *Supporting evidence*: Symptoms align with common chemotherapy side effects (nausea, vomiting, diarrhea).
   - *Contradictory evidence*: Absence of abdominal pain or other gastrointestinal distress.
2. **Postoperative/radiotherapy sequelae**
   - *Supporting evidence*: Altered bowel motility following rectal surgery.
   - *Contradictory evidence*: Atypical presentation (no persistent pain or obstructive symptoms).
3. **Gastrointestinal infection or other complications**
   - *Supporting evidence*: Persistent vomiting and diarrhea are suggestive of secondary pathology.
   - *Contradictory evidence*: No fever, hematochezia, or markers of severe infection.

Summary

A 46-year-old female with a history of rectal cancer surgery and postoperative chemotherapy presents with 20 days of frequent bowel movements, nausea, and occasional vomiting. Currently prescribed medications have provided partial symptom relief, but persistent bowel frequency significantly impacts daily functioning. The patient resides in an accessible environment but lacks psychological support. Further diagnostic evaluation (as above) is recommended to clarify etiology and guide targeted management.

Supplementary Table 4 (Continued) Example 2:

Patient Demographics

- **Sex**: Female
- **Age**: 36 years
- **Allergy History**: Unknown

Clinical Summary

- **Referred Department**: Endocrinology
- **Chief Complaint**: No overt physical discomfort.
- **History of Present Illness**: The patient has a history of diabetes mellitus and hyperthyroidism. Recent fasting blood glucose (FBG) measurement of 8 mmol/L indicates suboptimal glycemic control.
- **Past Medical History**: Diabetes mellitus, hyperthyroidism.

Symptomatology

- **Positive Symptoms**:
  - Elevated fasting blood glucose (8 mmol/L; normal range: 3.9–5.6 mmol/L).
- **Negative Symptoms**:
  - Absence of thirst, polyuria, fatigue, palpitations, or weight changes.
  - No headaches or depressive symptoms.
- **Ancillary Findings**:
  - FBG: 8 mmol/L (elevated).
  - No recent thyroid function tests (T3, T4, TSH).

Proposed Diagnostic Workup

1. **Glycated Hemoglobin (HbA1c)**:
   - *Rationale*: Assess long-term glycemic control and diabetes management efficacy.
2. **Thyroid Function Panel (T3, T4, TSH)**:
   - *Rationale*: Evaluate current thyroid status to guide hyperthyroidism management.
3. **Urinalysis**:
   - *Rationale*: Screen for renal complications, common in diabetic patients.

Differential Diagnoses

1. **Poorly Controlled Diabetes Mellitus**:
   - *Supporting Evidence*: Elevated fasting glucose (8 mmol/L).
   - *Contradictory Evidence*: Absence of classic hyperglycemic symptoms (e.g., polyuria, polydipsia).
2. **Hyperthyroidism (Graves' Disease):**
   - *Supporting Evidence*: History of hyperthyroidism with unmonitored thyroid hormone levels.
   - *Contradictory Evidence*: No overt symptoms (e.g., weight loss, palpitations).
3. **Diabetes-Hyperthyroidism Interplay**:
   - *Supporting Evidence*: Coexistence of both conditions, which may exacerbate metabolic dysregulation.
   - *Contradictory Evidence*: No clear symptomatic overlap is observed currently.

Summary

A 36-year-old female with a history of diabetes mellitus and hyperthyroidism presents with elevated fasting blood glucose (8 mmol/L), suggestive of suboptimal diabetes management. Despite the absence of typical symptoms (e.g., thirst, fatigue), thyroid function remains unassessed. Recommended investigations include HbA1c for glycemic control evaluation, thyroid function testing, and urinalysis to rule out renal complications. These steps will clarify disease activity and guide targeted therapeutic interventions.

Supplementary Table 4 (Continued) Example 3

Patient Demographics

- **Gender**: Male
- **Age**: 69 years
- **Allergy History**: Unknown

Clinical Information

- **Department**: Respiratory Medicine
- **Chief Complaint**: Dry cough persisting for over half a month, accompanied by dyspnea and chest tightness.
- **History of Present Illness**: Dry cough for 15 days; chest tightness and dyspnea.
- **Past Medical History**: Coronary heart disease (CHD), on regular medication but without routine follow-up.

Symptomatology

- **Positive Symptoms**:
  - Dry cough; chest tightness; dyspnea.
- **Negative Symptoms**:
  - No fever; no sputum production; no sore throat; no chest pain; no fatigue, weakness, or palpitations; no significant psychological distress.
- **Auxiliary Test Results**:
  - Unknown

Proposed Diagnostic Workup

1. **Chest X-ray**: To rule out pulmonary diseases or cardiac abnormalities.
2. **Electrocardiogram (ECG)**: Assess cardiac function and current status of CHD.
3. **Pulmonary Function Tests (PFTs)**: Evaluate lung function to detect COPD or other respiratory disorders.

Differential Diagnoses

1. **Chronic Obstructive Pulmonary Disease (COPD)**
   - **Supporting Evidence**: Symptoms align with COPD (dry cough, chest tightness, dyspnea).
   - **Contradictory Evidence**: No documented smoking history or exposure to irritants.

2. **Angina Pectoris Secondary to CHD**
    - **Supporting Evidence**: History of CHD; chest tightness may correlate with cardiac ischemia.
    - **Contradictory Evidence**: Absence of typical angina features (e.g., palpitations, exertion-related fatigue) and prolonged symptom duration.
3. **Upper Respiratory Tract Infection (URTI)**
    - **Supporting Evidence**: Dry cough could indicate viral infection.
    - **Contradictory Evidence**: Symptoms persist for 15 days without infectious signs (e.g., fever, sputum).

Summary Report
A 69-year-old male presents with dry cough, chest tightness, and dyspnea, alongside a history of CHD. Further diagnostic investigations (chest X-ray, ECG, PFTs) are recommended to clarify the etiology. Expedite evaluations and continue monitoring symptom progression. Immediate medical attention is advised if symptoms worsen or new manifestations arise.

Notes: The initial Chinese questionnaires have been translated into English and carefully reviewed to maintain their original meaning. Specifically, the principal investigator (SH) translated the Mandarin text into English. Two researchers (XT and SZ) compared the English and Chinese versions line by line to resolve semantic and cultural discrepancies.

Supplementary Table 5 The referral report evaluation rubrics

Citeria	Questions	Single Chocies
History taking	How do you rate the medical history documentation?	Very favorable, includes all critical and relevant information
		Favorable, includes critical historical information but misses some relevant details
		Neutral, includes critical historical information but lacks other important information
		Unfavorable, does not include critical historical information
		Very unfavorable, no content related to history taking
Diagnosis	How would you rate this physician's diagnosis?	Very favorable, includes the most likely diagnosis and all reasonable differential diagnoses
		Favorable, includes the most likely diagnosis and some reasonable differential diagnoses but misses closely related ones
		Neutral, includes the most likely diagnosis but lacks other reasonable differential diagnoses
		Unfavorable, does not include the most likely diagnosis but provides relevant content to help identify it
		Very unfavorable, no content related to the most likely diagnosis
Test ordering	How would you rate the tests and investigations recommended by this physician?	Very favorable, suggested tests are comprehensive and appropriate
		Favorable, suggested tests are not comprehensive but are appropriate
		Neutral, includes the most essential tests but also suggests inappropriate ones
		Unfavorable, does not include the most essential tests

		Very unfavorable, no content related to appropriate tests and investigations

Notes: The initial Chinese questionnaires have been translated into English and carefully reviewed to maintain their original meaning. Specifically, the principal investigator (SH) translated the Mandarin text into English. Two researchers (XT and SZ) compared the English and Chinese versions line by line to resolve semantic and cultural discrepancies.

Supplementary Table 6 A de-identified case of PreA interacting with real-world patients: The patient missed important information until later in the dialogue that required multi-disciplinary assessment

PreA: Hello, I'm your pre-specialist consultation assistant. You can communicate with me via text or voice messages.
Patient: I've been losing a lot of hair.
PreA: May I ask how old you are? And what is your gender?
Patient: Female, 54 years old.
PreA: Thank you for the information. How long has your hair loss been going on? Have you noticed any specific pattern of hair loss, such as localised hair loss or general hair loss?
Patient: It's all over my head. I also have trouble sleeping. The hair loss started suddenly; I used to lose much less hair before.
PreA: I see. You mentioned that the hair loss has suddenly worsened and is accompanied by poor sleep. Have you experienced significant stress or emotional changes recently? Such as stress related to work, family, or life?
Patient: No, none of these.
PreA: Alright, thank you for the additional information. Have there been any particular changes in your diet, such as changes in your diet structure or nutrient intake? Or have you taken any new medications or health supplements recently?
Patient: My diet is fine. It's just that before the Chinese New Year, I had a cold that lasted a long time. Then, when I sleep at night, I keep sweating; when I get up, my whole body is wet. Also, my ears have been ringing all the time. Sometimes I think it's the sound of water boiling, so I run over to check, but the water isn't boiling yet.
PreA: Thank you for your detailed description. You mentioned symptoms of a cold, night sweats, and tinnitus. I'd like to know if you have any other health problems, such as high blood pressure, diabetes, or other chronic diseases. Also, have you had any weight changes recently, or felt unusually fatigued?
Patient: None of those. I just sit at home all the time. I feel mentally tired, and sometimes extremely exhausted.
PreA: Thank you so much for your thoughtful responses. To sum up, it looks like you've been experiencing the following symptoms lately. Please don't hesitate to share these details openly with your specialist so they can best assist you.

Sudden worsening of general hair loss, poor sleep and night sweats, tinnitus, mental fatigue, and physical weakness.

Based on this information, I consider the following possible diagnoses:

Thyroid dysfunction (such as hyperthyroidism or hypothyroidism)
Supporting Evidence: Hair loss, fatigue, and excessive sweating can all be related to thyroid issues.
Opposing Evidence: Further tests are needed to confirm thyroid function..
Menopause-related symptoms
Supporting Evidence: Your age (54 years old) and night sweats may be related to hormonal changes.

Opposing Evidence: Tinnitus and fatigue are not necessarily directly related to menopause.
Chronic fatigue syndrome
Supporting Evidence: Long-term fatigue, poor sleep quality, accompanied by tinnitus.
Opposing Evidence: Lack of other characteristic symptoms.

Next, I suggest the following examinations to get a clearer and more accurate assessment:

Thyroid function tests (TSH, FT4, FT3): Given the issues of hair loss, fatigue, and sweating, checking thyroid function helps rule out thyroid diseases.
Blood routine examination: It can help detect problems such as anaemia or infection, which may be related to your overall health.
Hormone level tests: Checking hormone levels, such as estrogen, can provide a basis for screening menopause-related issues.

PreA: Please consider having these examinations to help with a clearer diagnosis and more effective treatment. If you have any other questions or concerns, feel free to ask—I'm here to help!
Patient: I've seen a dermatologist about the hair loss months ago. The dermatologist said it's seborrheic alopecia. My scalp used to itch a lot, and so did my body, but now it doesn't. The dermatologist advised me to visit the endocrinology department, but I have been busy with the Chinese New Year.
PreA: Thank you for providing this important information. You mentioned that the dermatology examination diagnosed seborrheic alopecia, and you previously had severe scalp and body itching, which has now subsided. Based on the additional information, I updated diagnostic considerations:

Seborrheic alopecia
Supporting Evidence: The dermatological diagnosis is consistent with your hair loss situation.
Opposing Evidence: A comprehensive diagnosis of seborrheic alopecia usually requires a combined endocrine assessment.
Endocrine disorders
Supporting Evidence: Endocrine issues may be related to worsened hair loss and physical fatigue, and may also explain your previous itching.
Opposing Evidence: There are no clear symptoms of endocrine disorders; further examinations are needed for confirmation.
Other skin problems
Supporting Evidence: In addition to seborrheic alopecia, there may be impacts from other skin problems. A combined assessment by the endocrinology and dermatology departments is recommended.
Opposing Evidence: You haven't mentioned neck symptoms or numbness.

Next, I suggest the following exams for a more precise assessment:

Endocrine tests (such as sex hormones, insulin, adrenal hormones, etc.): Your hair loss is related to endocrine factors, so it is necessary to check hormone levels.
Scalp examination by the dermatology department: To ensure that skin problems such as seborrheic dermatitis are evaluated and current symptoms are managed.
Blood glucose and blood lipid tests: There may be an association between metabolic diseases and hair loss, which needs further confirmation.

PreA: So now you are considering going to the endocrinology department for further consultation and examinations. If you have any other questions, I'll be happy to help answer them.
Patient: No more.
PreA: Alright, thank you for your cooperation and for sharing the information. I hope my suggestions are helpful to you. Please remember to seek further guidance from professional doctors. Wish you good health!
Patient: Thank you.

Note: Any advice rendered by PreA serves solely as a reference and should not be utilized as a definitive basis for disease therapy.

Notes: The initial Chinese questionnaires have been translated into English and carefully reviewed to maintain their original meaning. Specifically, the principal investigator (SH) translated the Mandarin text into English. Three researchers (XT, SZ, and SL) compared the English version and the Chinese version line by line to resolve semantic and cultural discrepancies.

Supplementary Table 7 A de-identified case of PreA interacting with real-world patients: The patient took medications without consulting a doctor

PreA: Hello, I'm your pre-specialist consultation assistant. You can communicate with me via text or voice messages.
Patient: I've had hand tremors and cramps since 2017.
PreA: Thank you for sharing this information. May I ask if you are male or female? Also, what is your current age?
Patient: Male, 30 years old.
PreA: Alright, you mentioned that you've had symptoms of hand tremors and cramps for many years. Could you describe the hand tremors and cramps in detail? For example, do the hand tremors occur continuously or occasionally? And in which parts of the body do the cramps mainly occur?
Patient: I don't know myself; people around me told me that I had convulsions.
PreA: I see, people around you observed that you have convulsions. You mentioned this has been going on for a long time. During this period, have you had any other related symptoms, such as fatigue, headaches, dizziness, or mood changes?
Patient: I have hand tremors, and I stay up late. My whole body becomes stiff, but I don't have headaches.
PreA: Thank you for providing detailed information. The hand tremors, general stiffness, and staying up late you mentioned are all important clues. Have you had any past medical history, such as neurological diseases, epilepsy, metabolic diseases (like diabetes), or other serious illnesses? Or do you have a family medical history?
Patient: My mother and grandmother both have epilepsy. My twin brother also has convulsions.
PreA: Thank you for providing detailed information. The family history of epilepsy and the fact that your twin brother also has convulsions make it more necessary for us to pay attention to your possible health issues. Are there any other influencing factors in your daily life, such as irregular diet, high stress, lack of exercise, etc.?
Patient: I'm under stress, but my diet is regular. Now I have social engagements, feel tired, and don't drink alcohol.
PreA: Thank you for sharing this information. You mentioned being under stress and feeling tired, but having a regular diet, which is very important. One last question: have you had problems with low mood, anxiety, or insomnia recently?
Patient: When I look at things, I feel dizzy, and my feet feel unsteady. I'm very dizzy.
PreA: Thank you for providing the symptom information. The dizziness when looking at things, unsteady feet, and severe dizziness you mentioned may be related to the previous symptoms. Based on our previous conversation, here are some possible diagnoses:

Epileptic seizures
Supporting Evidence: Family history of epilepsy, and you have convulsions.
Opposing Evidence: No clear reports of definite epileptic seizure symptoms over the years.
Anxiety disorder or stress-related illness
Supporting Evidence: Presence of stress and emotion-related issues, accompanied by dizziness.
Opposing Evidence: No clear manifestations of severe low mood and anxiety symptoms.

Metabolic or endocrine problems
Supporting Evidence: General stiffness and dizziness may be related to metabolic abnormalities (such as hypoglycemia).
Opposing Evidence: Regular diet and lack of relevant medical history.

Examinations are needed:

Electroencephalogram (EEG): It can detect brain activity and determine if there are signs of epileptic seizures.
Blood glucose level test: To rule out dizziness and stiffness caused by hypoglycemia
Routine blood test and biochemical examination: To check for metabolic and endocrine problems and ensure there are no other underlying health issues in the body.

Here's a quick summary for you to consider sharing clearly with your specialist:

Main complaints: Hand tremors, convulsions (known from bystanders), general stiffness, dizziness when looking at things, unsteady feet, and severe dizziness.
Medical history: Family history of epilepsy (mother, grandmother), and the twin brother has convulsions.
Living habits: Under stress, has social engagements, regular diet, but feels tired; does not drink alcohol.
Suggested further examinations: Including EEG, blood glucose level, and routine blood test to rule out related diseases.

PreA: I hope this information is helpful to you. I suggest going to the hospital for corresponding examinations as soon as possible, and taking appropriate treatment measures after a definite diagnosis. Please be sure to pay attention to changes in these symptoms and keep communicating. If you have other questions, please let me know.
Patient: What examinations do I need to do? I bought sodium valproate sustained-release tablets and took them by myself; do I still need to take them? Do I have epilepsy?
PreA: Based on the information and symptoms you provided, it is recommended that you undergo the following examinations:

Electroencephalogram (EEG): It can detect brain activity and determine if there are signs of epileptic seizures.
Blood glucose level test: To rule out dizziness and stiffness caused by hypoglycemia.
Routine blood test and biochemical examination: To check for metabolic and endocrine problems and ensure there are no other underlying health issues in the body.

PreA: Regarding sodium valproate sustained-release tablets, sodium valproate is a commonly used anti-epileptic drug, suitable for epileptic patients. If you are already taking this drug, be sure to have relevant examinations first, and then let professional doctors judge

whether to continue using it or adjust the dosage based on your examination results. If you have not been diagnosed with epilepsy, please do not decide to continue taking the drug on your own.

Please go to the hospital to consult a professional neurologist, discuss your symptoms and current medication situation, so as to conduct a detailed evaluation and formulate a correct treatment plan.

Do not arbitrarily use or stop taking the drug without clear advice from a doctor.

Please pay attention to changes in your symptoms and seek medical attention in a timely manner. If you have other questions, you can continue to ask.

Patient: I see.
PreA: Thank you very much for your cooperation. If you have any other questions or need further help, please feel free to let me know. I hope you can see a doctor as soon as possible, and I wish you a speedy recovery.
Patient: Thank you.

Note: Any advice rendered by PreA serves solely as a reference and should not be utilized as a definitive basis for disease therapy.

Notes: The initial Chinese questionnaires have been translated into English and carefully reviewed to maintain their original meaning. Specifically, the principal investigator (SH) translated the Mandarin text into English. Three researchers (XT, SZ, and SL) compared the English version and the Chinese version line by line to resolve semantic and cultural discrepancies.

Supplementary Table 8 A de-identified case of PreA interacting with real-world patients: The patient had concerns about examinations

PreA: Hello, I'm your pre-specialist consultation assistant. You can communicate with me via text or voice messages.
Patient: My head has been buzzing for a week.
PreA: May I ask if you are male or female? And how old are you?
Patient: 55 years old, male.
PreA: Thank you for the information. Could you describe the buzzing in the head in detail? For example, is it continuous or intermittent? Are there any accompanying symptoms such as headache, dizziness, tinnitus, or other discomforts?
Patient: It's continuous, and I have tinnitus. No headache, dizziness, or vomiting.
PreA: Got it, thank you for the detailed description. Have you recently had a cold, allergies, increased stress, or other health issues? In addition, do you have a history of diseases such as hypertension or diabetes?
Patient: I used to have atherosclerosis and frequent ventricular premature beats. No other diseases.
PreA: Thank you for your answer. Atherosclerosis and frequent ventricular premature beats are health issues that need attention. What's your living environment like? For example, are there any sources of noise? In your daily life, do you have habits like smoking, drinking alcohol, or other behaviors that may affect your health?
Patient: No noise. I smoke, but don't drink alcohol.
PreA: Thank you for the information. How often do you smoke? And about how long have you been smoking? Besides, do you engage in regular physical exercise?
Patient: One pack a day. Occasionally, take a walk.
PreA: Got it, thank you for sharing. How has your mental state been recently? Have you felt anxious, depressed, or had mood swings? This may also have some impact on your physical condition.
Patient: No mental problems.
PreA: Thank you for your reply. Based on our previous conversation, your described symptoms and medical history, I consider the following possible diagnoses:

Correlation between tinnitus and atherosclerosis
Supporting Evidence: The previous history of atherosclerosis may lead to insufficient blood supply to the ears.
Noise-induced tinnitus
Supporting Evidence: Your daily smoking habit may indirectly affect tinnitus.
Tinnitus caused by cardiovascular problems
Supporting Evidence: You have a history related to the heart; for example, ventricular premature beats may affect the circulatory system.

Recommended examinations:

Otolaryngology examination: To assess the source of tinnitus in detail, including hearing tests.
Electrocardiogram (ECG): To check for frequent ventricular premature beats and the electrical activity of the heart.
Blood pressure measurement and blood tests: To evaluate cardiovascular health and understand the extent of the impact of atherosclerosis.

PreA: Please consider undergoing the corresponding examinations based on this information. If you have any questions that need further discussion, please let me know at any time.
Patient: Do I need a brain examination?
PreA: In your case, brain examinations (such as head CT or MRI) are generally for patients with specific symptoms, such as new or sudden headaches, vision changes, severe dizziness, etc.
According to our previous conversation, you currently don't have these additional symptoms; the main issues are continuous tinnitus and a history related to atherosclerosis. Therefore, it may be more important to first undergo an otolaryngology examination and an electrocardiogram. However, if your doctor deems it necessary after evaluation, they may still recommend a brain examination.
Do you have any additional headaches or other neurological symptoms? If yes, a brain examination may be needed. If not, it's recommended to start with otolaryngology and cardiac evaluations.
Do you think this arrangement is appropriate?
Patient: I see.
PreA: Thank you for your understanding and cooperation. Please remember that it's very important to arrange the otolaryngology examination and electrocardiogram as soon as possible to find out the cause of tinnitus and your overall health status early. If you have any new symptoms or doubts, don't hesitate to get in touch with your doctor in time. Wish you a speedy recovery!
Patient: Alright.

Note: Any advice rendered by PreA serves solely as a reference and should not be utilized as a definitive basis for disease therapy.

Notes: The initial Chinese questionnaires have been translated into English and carefully reviewed to maintain their original meaning. Specifically, the principal investigator (SH) translated the Mandarin text into English. Three researchers (XT, SZ, and SL) compared the English version and the Chinese version line by line to resolve semantic and cultural discrepancies.

Clinical trial protocol and statistical analysis plan

Evaluating the effectiveness of large language models (LLM)-based health consultation models in improving patient experience and hospital operational efficiency

Principal investigators:

Dr. Shasha Han, PhD

Assistant Professor, Chinese Academy of Medical Sciences & Peking Union Medical College

Dr. Libing Ma, MD, PhD

Deputy Director, The First Affiliated Hospital of Guilin Medical University

Sponsor:

Dr. Shasha Han, PhD

Assistant Professor, Chinese Academy of Medical Sciences & Peking Union Medical College

Investigation sites:

The First Affiliated Hospital of Guilin Medical University, 1 Zhiyuan Road, Lingui District, Guilin, Guangxi, P. R. China

Affiliated Hospital of Gansu Medical College, 296 Eastern Kongtong Road, Kongtong District, Pingliang, Gansu, P. R. China

Trial registration: ChiCTR2400094159

Protocol version: V1.1

Funding: National Natural Science Foundation of China and Chinese Academy of Medical Sciences Innovation Fund. The sponsors and funders have no role in the study design, which includes the collection, management, analysis, and interpretation of data; the writing of the report; and the decision to submit the report for publication.

Table of Contents

Background and Objectives *4*

Methods *4*

Trial design 6

Participants 7

Interventions 7

Outcomes 8

Sample size 9

Randomization and blinding 9

Recruitment plan 10

Statistical methods 10

Ethics *10*

Good clinical practice 11

Protocol approval and amendments 12

Consent and assent 12

Confidentiality 13

Record retention 13

Dissemination *13*

References *13*

Background and Objectives

Large language models (LLMs) possess transformative potential to re-engineer patient clinical workflows and address systemic healthcare inefficiencies amplified by escalating demand. However, current applications remain largely confined to specific tasks, e.g., responding to patient portal messages [1 2], aiding physician clinical and management reasoning in experimental environments [3,4], or improving medical directions in online pharmacies[5] with limited integration into real-world clinical decision-making processes. Critically absent is evidence of LLMs operating within real-world clinical workflows: directly interfacing with socioeconomically diverse patient populations and augmenting physician judgment in high-volume settings.

Bridging this gap necessitates capabilities underexplored in prior research: (1) resolving the risks associated with developing LLMs using local natural primary care dialogues, which could codify and reinforce systemic deficits prevalent in resource-limited primary care settings, and (2) validating LLM-assisted clinical decision-making under real-world operational pressures. While localized dialogues, e.g., patient-nurse interactions in outpatient triage [6], young people and crisis responders' text conversations in mental health consultation [7], patient and doctor online medical conversations in telemedicine service [8], have informed niche LLM applications, their suitability for broader primary care deployment in resource-limited contexts remains contentious due to the potential for these data to mirror existing care deficits. Prior research has questioned the feasibility of relying solely on such data, instead favoring simulated dialogues derived from standardized

medical corpora[4,9], yet a comprehensive evaluation of the inherent risks of utilizing uncurated local data in clinical decision-making remains absent. This oversight carries profound implications: uncurated dialogues from resource-constrained settings could code systemic inequities, including simplified diagnostic heuristics, incomplete history-taking practices, and sociocultural biases, which LLMs risk perpetuating and disseminating at scale.[10,11] Notably, while the local data originates from local populations, no study has directly compared participatory co-design (actively embedding local stakeholders in model development) versus passive data harvesting (training on existing conversational corpora). This gap leaves unresolved whether LLMs should reflect local practices or reform them—a pivotal question for global health equity.

In this study, we aim to bridge the gaps in translating the potential of LLMs into tangible, real-world impacts, particularly within resource-constrained environments that connect primary and secondary care. We adopted a multi-stakeholder iterative co-design approach, engaging a diverse group of participants, including patients, care partners, community health workers, primary care physicians, specialist physicians, nurses, and hospital administrators. This collaborative effort focused on developing an LLM-based platform (OpenAI; GPT-4.0 mini) to facilitate primary care consultation and generate referral reports for assisting specialist physicians. We evaluated the potential negative impact of uncurated local natural language data on the performance of the co-designed LLM through interactions with simulated patients. Subsequently, we validated the co-designed chatbot in a multi-center pragmatic randomized controlled trial (RCT) involving 24

medical disciplines, demonstrating its ability to enhance both operational efficiency and the humanistic dimensions of care delivery in real-world settings.

Objectives

To investigate whether using the PreA for pre-specialist consultation improves operational efficiency and the humanistic dimensions of care delivery in real-world settings, and whether the PreA can operate independently without a medical assistant.

Hypotheses

Compared to the No-PreA group, the PreA will demonstrate enhanced operational efficiency and the humanistic dimensions of care delivery. The PreA operates independently without needing additional workforce.

Methods

Trial design

We will conduct a parallel randomized controlled trial. Participants are randomly assigned to one of three groups: (1) the PreA-only group, where they initially interacted with the PreA via mobile phone before their physician consultation; (2) the PreA-human group, where they initially interacted with the PreA via mobile phone with the guidance of a medical assistant before their physician consultation; and (3) the control group, receiving standard physician-only care (No-PreA). The allocation ratio is 1:1:1. The PreA-human arm is

included to investigate the potential need for human augmentation in the implementation of PreA. Consequently, the primary comparison is between the PreA-only arm and the No-PreA control, with a secondary comparison of the PreA-only arm and the PreA-human arm. We will record the entire dialogue process and gather participant feedback to evaluate the quality of the dialogue. Reporting will adhere to the CONSORT-AI guidelines. [12]

Patients, caregivers, community health workers, primary care physicians, specialist physicians, nurses, and other stakeholders have been involved in the design of the interventions and the trial through the co-design process.

Participants

Inclusion Criteria: Participants must demonstrate a need for health consultation or express a willingness to engage in PreA health consultations. Inclusion criteria were as follows: (1) aged between 20 years and 80 years; (2) visit the particpating physicians at the study medical centers; (3) eligible for communicative interaction via mobile phone; (4) eligible to complete the post-consultation questionaries; and (5) have signed informed consent.

Exclusion Criteria: (1) the presence of psychological disorders; (2) any other medical events that are determined ineligible for LLMs-based conversation; and (3) refusal to sign informed consent.

Interventions

Participants are randomly assigned to one of three study arms: (1) the PreA-only group, where they initially interact with the PreA via mobile phone before their physician

consultation; (2) the PreA-human group, where they initially interact with the PreA via mobile phone with the guidance of a medical assistant before their physician consultation; and (3) the control group, receiving standard physician-only care (No-PreA). In the PreA-human group, participants are informed that PreA functions similarly to dialogue on WeChat. They are also provided with technical support for using PreA. Conversely, participants in the PreA-only group will not receive any technical assistance. In both the PreA-only and PreA-human groups, a referral report generated from the PreA is provided to the physicians before any face-to-face interaction with the participant. For the No-PreA group, the referral report includes only the patient's sex and age. Physicians are requested to review the referral report before providing care to patients and rate its value in facilitating patient care using a five-point Likert scale. After the consultations, patients and care partners complete a post-consultation questionnaire to reflect on their experiences. Additionally, physicians complete a feedback questionnaire at the conclusion of their shifts.

Outcomes

The primary outcomes are the duration of physician-patient consultations, physician perception of primary-secondary care coordination, and patient perception of ease of communication. Secondary outcomes included: (1) physician workload (measured by number of patients per shift); (2) patient perceived physician attentiveness during visits, patient satisifaction, interpersonal regard, and future acceptability; (3) physician experience with perceived utility of PreA, ease of communication ease with patients, and

relief of workload; (4) physician clinical decision-making (analyzing clinical note content). Consultation duration, number of patients per shift, and clinical notes will be retrieved from the PreA platform and electronic hospital records. Physician-perceived care coordination will be assessed through physicians' evaluations of the helpfulness of referral reports in facilitating patient care, utilizing a five-point Likert scale. Patient perception of ease of communication and other self-reported outcomes will be measured using a post-visit questionnaire based on five-point Likert scales.

Sample size

The sample size was estimated to depend upon the primary comparison, PreA-only versus No-PreA. The target minimum sample size of 2010 participants (670 participants per study arm) was prespecified based on a power analysis using the preliminary data of 90 patients in the pilot study before study enrollment. This minimum target sample size ensured sufficient power (>80%) for the primary outcome at a significance level of 0.05.

Randomization and blinding

We use individual-level parallel randomization without stratification, utilizing a computer-generated random sequence for participant assignment to each experimental group. We will implement allocation concealment to maintain the confidentiality of the random allocation and minimize bias. This trial is single-blinded: While the patients know their group assignments, the physicians are uninformed about the PreA-intervention groups (PreA-only or PreA-human), and the researchers are also unaware of the assignments.

Recruitment plan

In the trial, we will recruit physicians across diverse medical disciplines from the two medical centers. The clinical research team will proactively contact potential adult patients from the waiting room who are scheduled to see the participating physicians. For pediatric patients and adult patients who do not have a smartphone, the clinical research team contacts their caregivers. For those who express interest, the research team will provide comprehensive descriptions of the study, emphasizing that it is exploratory and that any advice provided by PreA serves solely as a reference and should not be used as a definitive basis for disease therapy. Patients and caregivers will receive an informed consent form before enrollment and will have the opportunity to ask questions. After this process, potential patients and caregivers who meet the established inclusion and exclusion criteria will be formally recruited.

Statistical methods

<u>Analysis of healthcare delivery</u>

To ensure objectivity, data collection and subsequent statistical analyses will be conducted by independent researchers. We will evaluate covariate balancing across three groups using the ANOVA test for continuous variables and the Chi-squared test for categorical variables. We will assess the normality of scale value distributions and use two-sample t-tests with unequal variances for intergroup comparisons where appropriate. For dimensions that exhibited significant skewness, we employ non-parametric Mann-Whitney U tests. All statistical tests are two-tailed, with a significance threshold set at P <

0.05. Additionally, we apply the Benjamini-Hochberg adjustment for multiple testing corrections based on the total number of tests performed.[13] We estimate the relative difference between PreA-only and the No-PreA group as the difference in means divided by the means of the No-PreA group. Secondary analyses involve a focused assessment of the consistency of the findings across diverse socioeconomic and clinical subgroups.

Analysis of clinical notes

To investigate the effectiveness of PreA-assisted physician consultation on clinical decision-making, we will conduct a classification analysis comparing clinical notes from PreA-assisted arms (PreA-only and PreA-human groups) against those from the standard arm (No-PreA). To evaluate domain-specific variations in clinical notes between groups, we will conduct a comprehensive analysis of clinical documentation across five standardized domains: history-taking, physical examination, diagnosis, test ordering, and treatment plans.

Ethics

Good clinical practice

This study will be conducted in accordance with the highest ethical and scientific standards, adhering to Good Clinical Practice (GCP) guidelines (ICH E6 Guideline for Good Clinical Practice, 1 May 1996), the Declaration of Helsinki, and relevant local regulations.

Protocol approval and amendments

Before initiating a research study, the principal investigators will seek approval from the ethics committee for the research protocol, informed consent procedures, and any other written materials that will be provided to study participants. Throughout the study, particularly at critical junctures or when significant changes occur, the principal investigators will proactively update the ethics committee. The principal investigators will ensure that any amendments to the protocol or informed consent documents receive approval from the ethics committee prior to implementation, except in situations where immediate changes are essential to safeguard participant safety. In such instances, they will notify the ethics committee as promptly as possible.

Consent and assent

The investigators will ensure that study participants, or their legal representatives, fully understand the study and will provide written informed consent. Participants will receive ample time to consider their participation and will be instructed on how to sign the informed consent electronically. They will not be enrolled until they sign the informed consent. Throughout the study, participants will receive updated versions of the informed consent document and other written materials as they become available. The investigator will retain the informed consent documents.

Confidentiality

In the collection and use of data for this study, the investigators will implement stringent data processing and anonymization procedures. The data will be used exclusively for research purposes and will be strictly anonymized to minimize the risk of identifying individual participants. Additionally, throughout the study, the investigators will apply robust data privacy measures to safeguard the confidentiality of personal information further, ensuring that the integrity and security of the data remain uncompromised.

Record retention

Data retention will last for 10 years following the completion of the study.

Dissemination

The principal investigators commit to communicating the results of this study and making them publicly available. Public disclosure includes publishing a paper in a scientific journal, submitting an abstract for a poster or oral presentation at a scientific meeting, or other means of disclosure. No confidential information will be disclosed.

References

1. Ayers JW, Poliak A, Dredze M, et al. Comparing physician and artificial intelligence chatbot responses to patient questions posted to a public social media forum. *JAMA Intern Med*. 2023;183(6):589-596.

2. Chen S, Guevara M, Moningi S, et al. The effect of using a large language model to respond to patient messages. *Lancet Digit Health*. 2024;6(6):e379-e381.

3. Goh E, Gallo RJ, Strong E, et al. GPT-4 assistance for improvement of physician performance on patient care tasks: a randomized controlled trial. *Nature Medicine 2025*. Published online February 5, 2025:1-6.

4. Tu T, Schaekermann M, Palepu A, et al. Towards conversational diagnostic artificial intelligence. *Nature 2025*. Published online April 9, 2025:1-9.

5. Pais C, Liu J, Voigt R, Gupta V, Wade E, Bayati M. Large language models for preventing medication direction errors in online pharmacies. *Nature Medicine 2024 30:6*. 2024;30(6):1574-1582.

6. Wan P, Huang Z, Tang W, et al. Outpatient reception via collaboration between nurses and a large language model: a randomized controlled trial. *Nature Medicine 2024 30:10*. 2024;30(10):2878-2885.

7. Obadinma S, Lachana A, Norman ML, et al. The FAIIR conversational AI agent assistant for youth mental health service provision. *npj Digital Medicine 2025 8:1*. 2025;8(1):1-13.

8. Li Y, Li Z, Zhang K, Dan R, Jiang S, Zhang Y. ChatDoctor: A Medical Chat Model Fine-Tuned on a Large Language Model Meta-AI (LLaMA) Using Medical Domain Knowledge. *Cureus*. 2023;15(6):e40895.

9. McDuff D, Schaekermann M, Tu T, et al. Towards accurate differential diagnosis with large language models. *Nature 2025*. Published online April 9, 2025:1-7.

10. Clusmann J, Kolbinger FR, Muti HS, et al. The future landscape of large language models in medicine. *Communications Medicine 2023 3:1*. 2023;3(1):1-8.

11. Hager P, Jungmann F, Holland R, et al. Evaluation and mitigation of the limitations of large language models in clinical decision-making. *Nature Medicine 2024 30:9*. 2024;30(9):2613-2622.

12. Liu X, Cruz Rivera S, Moher D, et al. Reporting guidelines for clinical trial reports for interventions involving artificial intelligence: the CONSORT-AI extension. *Nat Med*. 2020;26(9):1364-1374.

13. Krzywinski M, Altman N. Points of significance: Comparing samples-part II. *Nat Methods*. 2014;11(4):355-356.

研究方案和数据分析计划

评估健康咨询大模型在现实世界中的可用性与有效性的随机对照临床试验研究

方案版本：V1.1

版本日期：2025 年 1 月 30 日

项目负责人：韩莎莎，中国医学科学院北京协和医学院，群医学及公共卫生学院

马礼兵，呼吸与危重症医学科，桂林医学院第一附属医院

项目资助情况：国自然科学基金和中国医学科学院创新工程基金

一、研究背景和意义

大型语言模型（LLMs）具有重塑患者临床工作流程，解决因医疗健康需求不断升级而加剧的系统性医疗效率低下问题的变革性潜力。然而，目前的应用仍主要局限于特定任务，例如，回应患者门户消息[1,2],、在实验环境中协助医生管理推理过程[3,4]，或在在线药房中改善医疗指导[5]，且与实际临床决策过程的整合有限。这方面，关键是缺乏 LLMs 在真实临床工作流中运行的证据：它们是否能直接与社会经济背景多样的患者群体互动，以及在高容量环境中辅助医生判断。

弥合这一差距需攻克既往研究中未充分探索的关键问题：(1) 解决直接使用本地自然初级医疗对话开发大型语言模型所带来的风险，此类对话数据可能会编码并强化资源有限的初级保健环境中普遍存在的系统性缺陷；(2) 在真实世界运营和时间压力下验证 LLM 辅助临床决策的有效性。虽然本地化对话，例如，门诊分诊中的患者与护士互动[6]，心理健康咨询中青少年与危机干预者的文本交流[7]，远程医疗服务中的患者与医生在线对话[8]，已支撑特定场景的LLM

应用，但因这些数据可能复现现有诊疗场景的缺陷，其在资源受限地区广泛部署的适用性仍存争议。

既往研究对仅依赖此类数据的可行性提出了质疑，转而更倾向于来自标准化医疗语料库的模拟对话；[4,9] 但对在临床决策中使用未经筛选的本地数据所潜在风险的全面评估仍然缺失。这一疏漏具有深远的影响：来自资源有限环境的未经筛选的对话可能会固化系统性不平等，包括简化的诊断启发法、不完整的病史采集以及社会文化偏见，而大型语言模型有可能在大规模传播中延续和放大这些偏见。[10,11] 值得注意的是，虽然本地数据来源于本地人群，但尚无研究直接比较参与式协同设计（即让本地利益相关者参与模型开发）与被动数据采集（即在现有对话语料上训练）之间的差异。这一空白导致 LLMs 应反映本地实践还是改革实践的核心问题悬而未决——这恰是实现全球健康公平的关键问题。

本研究将旨在弥合将大型语言模型潜力转化为切实的实际应用的差距，特别是在连接基层和二级医疗的资源有限环境中。本研究采用多方利益相关者的迭代共设计方法，整合患者、照护者、社区卫生工作者、基层医生、专科医生、护士及医院管理者等多方参与者，共同开发基于 LLM（OpenAI GPT-4.0 mini）的初级诊疗咨询及专科转诊报告生成平台，预咨询 PreA（pre-assessment）智能咨询系统。通过与模拟患者的互动，评估未经筛选的本地自然语言数据对共同设计的 LLM 性能可能产生的负面影响；继而在涵盖 24 个医学学科的多中心实用随机对照试验（RCT）中验证了该 PreA 智能咨询系统，证明其在提升运营效率和改善照护人文关怀方面在实际环境中的应用潜力。

二、研究目的

本研究目的是评估在真实医疗场景中应用 PreA 智能咨询系统于专科接诊前环节，是否既能提升诊疗运行效率与照护人文关怀维度，又可实现无医助独立运作。

三、研究假设

相较于无 PreA 组，PreA 系统将显著优化诊疗效率与人本关怀指标，且无需额外人力支持即可独立运行。

四、研究方法

1. 研究设计

本研究采用平行式随机对照试验设计。在桂林医学院第一附属医院、甘肃医学院附属医院均按照统一标准和流程开展研究。参与者将被1:1:1随机分配至以下三组之一：(1) 纯PreA组：在医生问诊前，通过手机与PreA智能咨询系统进行初步交互；(2) PreA人工辅助组：在医生问诊前，由人工助理指导通过手机与PreA智能咨询系统交互；(3) 对照组：接受仅由医生进行的标准诊疗（无PreA干预）。设置PreA人工辅助组旨在探究PreA应用中是否需要人工辅助支持。因此，主要比较将分析纯PreA组与无PreA对照组，次要比较将分析纯PreA组与PreA人工辅助组的差异。完整的对话过程和参与者的反馈将被记录下来，用以判断对话的质量。将按照CONSORT-AI指南规范[12]，对研究进行报告。

患者、家属、社区卫生工作人员、基层医生、专科医生、护士、及其他相关利益人通过协同设计流程全程参与了干预方案及试验设计。

2. 研究参与者

2.1 研究纳入标准

(1) 年龄20–80周岁；(2) 在研究医疗中心接受参与研究的医生接诊；(3) 具备通过手机进行有效交流的能力；(4) 可完成诊后问卷调查；(5) 已签署知情同意书。

2.2 研究排除标准

有心理障碍或其他可能影响沟通交流或评估完整性的个体；拒绝签署知情同意书。

3. 研究流程

参与者进入该研究项目将被随机分配至三组之一：(1)纯PreA组：通过手机在医生接诊前与PreA智能咨询系统交互；(2)PreA人工辅助组：在人工助理指导下通过手机在医生接诊前与PreA智能咨询系统交互；(3)对照组：接受医生标准诊疗（无PreA）。PreA人工辅助组受试者将被告知PreA智能咨询系统交互模式类似微信对话并可以获得技术协助，而纯PreA组不提供技术协助。两组PreA干预组在医患面谈前均会向医生提供PreA生成的转诊报告；对照组转诊报告仅含患者性别与年龄。医生需在接诊前审阅报告，并使用五点李克特量表评估其辅助诊疗价值。 接诊后患者及陪护人员需完成诊后体验问卷，医生则需在当班结束时填写反

馈问卷。

4. 主要测量指标或结局指标的选择和确认

主要结局指标为医患就诊时长（Duration of physician-patient consultations）、基层-专科医疗协同度（Physician perception of primary-secondary care coordination）、患者感知的沟通顺畅度 （Patient perception of ease of communication）。

次要结局指标包括医生工作负荷（通过每班接诊患者数量化）、患者感知的就诊关注度（Physician attentiveness）、满意度（Patient satisfaction）、医患关系认同度（Interpersonal regard）及未来接受度（Future acceptability）；医生体验的 PreA 效用度（Perceived utility of PreA）、患者沟通顺畅度（Ease of communication ease with patients）、工作负荷缓解度 (Relief of workload); 医生临床决策行为（Clinical decision-making; 通过分析病例记录观察）。

就诊时长、医生每班接诊量和病例记录从 PreA 平台及医院电子健康档案系统（EHR）自动提取。医疗协同度和其他医生体验相关的次要结局由医生通过五点李克特量表由医生自我报告；患者感知的沟通顺畅度和其他患者感知的次要结局由患者通过五点李克特量表由患者自我报告。

5. 受试者招募

在本试验中，我们将从两家医疗中心招募多学科医师参与研究。临床研究团队将主动接洽候诊区预约参与研究医师的潜在成年患者。针对儿科患者及无个人智能手机的成年患者，研究团队将联系其照护者（含监护人/陪同人员）。对表示参与意向者，研究团队将详细说明研究目的、流程等信息，并特别强调本研究属探索性试验，PreA 提供的建议仅供参考不应作为疾病治疗的确切依据。患者及照护者在入组前将签署知情同意书，并可随时提出质询。完成上述流程后，符合既定纳入排除标准的潜在受试者将被正式纳入研究。

6. 随机化分组和盲法

本实验采用面向个体平行随机设计，无分层。使用计算机软件生成一个随机序列。参与者将根据该随机序列被分配到每个实验组。本实验将实施分配隐藏策略，以确保随机分配的保密性和减少偏倚。 分组信息及组别操作材料在干预实施前不会对任何参与者或项目实施人员透

露。本试验为单盲设计：患者知晓分组情况；医师不知晓 PreA 干预组别（纯 PreA 组或 PreA 人工辅助组）；研究人员亦不知晓分组信息。

在本研究中，将对结局评估者和数据分析者实施盲法，以确保研究的客观性和结果的有效性。

五、数据分析计划

1. 样本量的计算和推理

本研究样本量基于主要比较组（纯 PreA 组 vs 无 PreA 对照组）计算。根据入组前预试验 90 例患者的初步数据实施把握度分析，预先设定最小目标样本量为 2010 例（每组 670 例）。该样本量可在显著性水平 $\alpha=0.05$ 时，为主要结局指标提供>80%的统计有效度。

2. 数据分析

医疗照护实施效能分析

为确保客观性，数据收集及后续统计分析将由独立研究人员执行。采用 ANOVA 检验评估三组连续变量的协变量平衡性，卡方检验评估分类变量。采用双样本 t 检验（并考虑不等方差的情况）进行组间比较，检验尺度值分布正态性，若存在显著偏态则改用非参数 Mann–Whitney U 检验。所有检验均为双尾检验（显著性阈值 $P<0.05$），并基于总检验次数进行 Benjamini–Hochberg 多重检验校正。[13] 主要分析中，通过（纯 PreA 组均值–无 PreA 组均值）/无 PreA 组均值计算组间相对差异；次要分析将重点评估不同社会经济及临床亚组中研究结果的一致性。

临床文档分析

为探究 PreA 智能咨询系统辅助诊疗对临床决策的影响，我们将开展分类对比分析：比较 PreA 辅助组（含纯 PreA 组及 PreA 人工辅助组）与标准组（无 PreA 组）的临床记录；并通过病史采集、体格检查、诊断结论、检验医嘱及治疗方案五大标准化领域，系统分析组间临床文档的差异。

六、道德伦理

1. 临床试验规范

本研究将遵循最高伦理与科学标准，严格执行 《药物临床试验质量管理规范》(ICH E6，1996 年 5 月 1 日版)、《赫尔辛基宣言》及相关相关法规要求。

2. 伦理委员会

在研究开始之前，研究者应获得伦理委员会对研究方案、知情同意书以及需要提供给研究参与者的其它书面信息的批准。在研究中止和/或完成时，研究者必须书面通知伦理委员会；研究者必须及时向伦理委员会报告所有研究工作中发生的变化（如方案和/或知情同意书的修订），并且在未获得伦理委员会批准之前不得执行这些变动，除非是为了消除对研究参与者明显且直接的风险而做出的变更，在发生这类情况时，将通知伦理委员会。

2. 参与者信息及知情同意

研究者必须向研究参与者或其监护人提供易于理解的并且经伦理委员会批准的知情同意书，并给与研究参与者或其监护人充分的时间考虑本项研究，在获得研究参与者签署的书面知情同意书之前，研究参与者不得入组。在研究参与者参与期间，将向研究参与者提供所有更新版本的知情同意书以及书面信息。知情同意书应作为临床研究的重要文档保留备查。

七、保密及数据安全

本试验数据收集中，数据将进行脱敏、去标识化处理。数据仅用于课题研究，且在研究过程中采用严格保密措施，以尽可能降低识别主体身份的风险，避免对数据信息主体产生不必要的风险。

八、研究结果的发布方式

本研究主要研究者承诺将及时公布研究结果并向公众公开。公开方式包括在科学期刊发表学术论文，向学术会议提交摘要，和其他适宜的公开途径。所有披露内容均不包含任何保密信息。

九、参考文献

1. Ayers JW, Poliak A, Dredze M, et al. Comparing physician and artificial intelligence chatbot responses to patient questions posted to a public social media forum. *JAMA Intern Med*. 2023;183(6):589-596.
2. Chen S, Guevara M, Moningi S, et al. The effect of using a large language model to respond to patient messages. *Lancet Digit Health*. 2024;6(6):e379-e381.

3. Goh E, Gallo RJ, Strong E, et al. GPT-4 assistance for improvement of physician performance on patient care tasks: a randomized controlled trial. *Nature Medicine 2025*. Published online February 5, 2025:1-6.
4. Tu T, Schaekermann M, Palepu A, et al. Towards conversational diagnostic artificial intelligence. *Nature 2025*. Published online April 9, 2025:1-9.
5. Pais C, Liu J, Voigt R, Gupta V, Wade E, Bayati M. Large language models for preventing medication direction errors in online pharmacies. *Nature Medicine 2024 30:6*. 2024;30(6):1574-1582.
6. Wan P, Huang Z, Tang W, et al. Outpatient reception via collaboration between nurses and a large language model: a randomized controlled trial. *Nature Medicine 2024 30:10*. 2024;30(10):2878-2885.
7. Obadinma S, Lachana A, Norman ML, et al. The FAIIR conversational AI agent assistant for youth mental health service provision. *npj Digital Medicine 2025 8:1*. 2025;8(1):1-13.
8. Li Y, Li Z, Zhang K, Dan R, Jiang S, Zhang Y. ChatDoctor: A Medical Chat Model Fine-Tuned on a Large Language Model Meta-AI (LLaMA) Using Medical Domain Knowledge. *Cureus*. 2023;15(6):e40895.
9. McDuff D, Schaekermann M, Tu T, et al. Towards accurate differential diagnosis with large language models. *Nature 2025*. Published online April 9, 2025:1-7.
10. Clusmann J, Kolbinger FR, Muti HS, et al. The future landscape of large language models in medicine. *Communications Medicine 2023 3:1*. 2023;3(1):1-8.
11. Hager P, Jungmann F, Holland R, et al. Evaluation and mitigation of the limitations of large language models in clinical decision-making. *Nature Medicine 2024 30:9*. 2024;30(9):2613-2622.
12. Liu X, Cruz Rivera S, Moher D, et al. Reporting guidelines for clinical trial reports for interventions involving artificial intelligence: the CONSORT-AI extension. *Nat Med*. 2020;26(9):1364-1374.
13. Krzywinski M, Altman N. Points of significance: Comparing samples-part II. *Nat Methods*. 2014;11(4):355-356.
