# Supplementary material for: An LLM chatbot to facilitate primary-to-specialist care transitions: a randomized controlled trial
Source: Nat Med. 2026 Jan 19;32(3):934–42. doi: 10.1038/s41591-025-04176-7 (PMC13004692; doi:10.1038/s41591-025-04176-7)
Supplement: Supplementary file 2 — Reporting Summary [file 41591_2025_4176_MOESM2_ESM.pdf]

nature portfolio

Corresponding author(s): Shasha Han

Last updated by author(s): Oct 22, 2025

Reporting Summary

Nature Portfolio wishes to improve the reproducibility of the work that we publish. This form provides structure for consistency and transparency in reporting. For further information on Nature Portfolio policies, see our Editorial Policies and the Editorial Policy Checklist.

Statistics

For all statistical analyses, confirm that the following items are present in the figure legend, table legend, main text, or Methods section.

n/a	Confirmed	
☐	☒	The exact sample size (*n*) for each experimental group/condition, given as a discrete number and unit of measurement
☐	☒	A statement on whether measurements were taken from distinct samples or whether the same sample was measured repeatedly
☐	☒	The statistical test(s) used AND whether they are one- or two-sided<br>*Only common tests should be described solely by name; describe more complex techniques in the Methods section.*
☐	☒	A description of all covariates tested
☐	☒	A description of any assumptions or corrections, such as tests of normality and adjustment for multiple comparisons
☐	☒	A full description of the statistical parameters including central tendency (e.g. means) or other basic estimates (e.g. regression coefficient) AND variation (e.g. standard deviation) or associated estimates of uncertainty (e.g. confidence intervals)
☐	☒	For null hypothesis testing, the test statistic (e.g. *F*, *t*, *r*) with confidence intervals, effect sizes, degrees of freedom and *P* value noted<br>*Give P values as exact values whenever suitable.*
☒	☐	For Bayesian analysis, information on the choice of priors and Markov chain Monte Carlo settings
☒	☐	For hierarchical and complex designs, identification of the appropriate level for tests and full reporting of outcomes
☒	☐	Estimates of effect sizes (e.g. Cohen's *d*, Pearson's *r*), indicating how they were calculated

Our web collection on statistics for biologists contains articles on many of the points above.

Software and code

Policy information about availability of computer code

Data collection	Our co-designed GPT-4-powered PreA platform (OpenAI; GPT-4o mini) chatbot was used to collect coversational and survey data.
Data analysis	Comparative statistical analyses were detailed in the paper. Python 3.7 and R 4.3.0 were used to perform the statistical analyses and present the results. Code for classification analysis and data visualization can be found at the following link (https://github.com/ShashaHan-collab/PreA-OutpatientRCT).

For manuscripts utilizing custom algorithms or software that are central to the research but not yet described in published literature, software must be made available to editors and reviewers. We strongly encourage code deposition in a community repository (e.g. GitHub). See the Nature Portfolio guidelines for submitting code & software for further information.

Data

Policy information about availability of data

All manuscripts must include a data availability statement. This statement should provide the following information, where applicable:

- Accession codes, unique identifiers, or web links for publicly available datasets
- A description of any restrictions on data availability
- For clinical datasets or third party data, please ensure that the statement adheres to our policy

The study protocol is provided in the Supplementary Information. Source data are provided in Tables and Extended Data Tables and can be accessed via the code repository (https://github.com/ShashaHan-collab/PreA-OutpatientRCT). Raw conversation data are not publicly available due to the need to protect participant

privacy, in accordance with the ethical approval for this study. Anonymized, non-dialogue individual-level data underlying the results can be requested by qualified researchers for academic use. Requests should include a research proposal, statistical analysis plan, and justification for data use, and can be submitted via email to S.H. (hanshasha@pumc.edu.cn). All requests will be reviewed by the Chinese Academy of Medical Sciences & Peking Union Medical College and the ethics committee of the First Affiliated Hospital of Guilin Medical University. Applicants will receive an initial response within two months, and approved requests will be granted access via a secure platform after execution of a data access agreement.

Research involving human participants, their data, or biological material

Policy information about studies with human participants or human data. See also policy information about sex, gender (identity/presentation), and sexual orientation and race, ethnicity and racism.

Reporting on sex and gender

Our trial analysis included 2,069 participants (691 with PreA-only, 689 with PreA-human, and 689 with No-PreA), with a mean age of 47.6 (SD 14.6), and 1,141 women (55.1%) and 928 men (44.9%).

Reporting on race, ethnicity, or other socially relevant groupings

The majority of participants (1,620, 78.3%) were patients themselves, while the remainder were the patients' care partners. Less than half (881, 42.6%) of participants were currently unemployed or retired, and 770 (37.2%) of participants had an income (including income from an office, employment on a full-time, part-time, or casual basis, or a pension from former employment) of less than 2000 RMB per month. 313 (15.1%) participants had education attainment less than primary school or below, 1073 (51.9 %) participants had high school, and 683 (33.0%) had college or above.

First, the generalizability of our time-reduction findings may be context-dependent, as our study was conducted in high-volume, resource-constrained hospital settings. The effectiveness of PreA is intrinsically tied to this environment of high clinical demand and standardized workflows, and validation in diverse healthcare systems is warranted. Second, the single-blinded, pragmatic trial design, while reflecting real-world conditions where patients would naturally know their pre-consultation experience, introduces potential performance bias as patients were aware of their group assignment. However, several factors mitigate this concern: the concordance of findings across multiple outcome assessments, the absence of significant differences in clinical documentation between groups, and the alignment of control group consultation times with established practice patterns.

Population characteristics

Our trial analysis included 2,069 participants (691 with PreA-only, 689 with PreA-human, and 689 with No-PreA), with a mean age of 47.6 (SD 14.6), and 1,141 women (55.1%) and 928 men (44.9%). The majority of participants (1,620, 78.3%) were patients themselves, while the remainder were the patients' care partners. Less than half (881, 42.6%) of participants were currently unemployed or retired, and 770 (37.2%) of participants had an income (including income from an office, employment on a full-time, part-time, or casual basis, or a pension from former employment) of less than 2000 RMB per month. 313 (15.1%) participants had education attainment less than primary school or below, 1073 (51.9 %) participants had high school, and 683 (33.0%) had college or above. 1186 (57.3%) of participants consulted for medical specialty, 558 (27.0%) for surgical, 194 (9.4%) for a specialty that provides both medical and surgical treatments, and the remaining consulted for pediatrics. These baseline covariates were well-balanced across the three intervention groups, with no statistically significant differences in covariate distributions (Table 1).

Recruitment

In the trial, we recruited 111 physicians across 24 medical disciplines (Supplementary Table 7) from the two medical centers. The clinical research team proactively contacted potential adult patients from the waiting room who were scheduled to see the participating physicians. For pediatric patients and adult patients who did not have a smartphone, the clinical research team contacted their caregivers. For those who indicated interest, the research team provided comprehensive descriptions of the study, emphasizing that it is exploratory and that any advice rendered by PreA serves solely as a reference and should not be utilized as a definitive basis for disease therapy. Patients and caregivers received an informed consent form before enrollment and will have the opportunity to ask questions. After this process, potential patients and caregivers who met the established inclusion and exclusion criteria were formally recruited.

Ethics oversight

The Chinese Academy of Medical Sciences & Peking Union Medical Colleges and the local medical ethics committee of the First Affiliated Hospital of Guilin Medical University approved the study. The institutional review boards of the Affiliated Hospital of Gansu Medical College approved the study protocol based on their review and the approval from the medical ethics committee of the First Affiliated Hospital of Guilin Medical University. The trial followed the Declaration of Helsinki and the International Conference of Harmonization Guidelines for Good Clinical Practice. We obtained informed consent from all participants (physicians, patients, and caregivers) in this study. All participants were informed that this was an exploratory experiment, and the results should not be interpreted as direct guidance for clinical interventions at this stage. This study implemented stringent data protection measures, ensuring that all data were anonymized and encrypted to protect privacy. This trial is registered at the Chinese Clinical Trial Registry (identifier: ChiCTR2400094159). The trial protocol and statistical analysis plan were provided in the Supplementary Materials.

Note that full information on the approval of the study protocol must also be provided in the manuscript.

Field-specific reporting

Please select the one below that is the best fit for your research. If you are not sure, read the appropriate sections before making your selection.

☒ Life sciences ☐ Behavioural & social sciences ☐ Ecological, evolutionary & environmental sciences

For a reference copy of the document with all sections, see nature.com/documents/nr-reporting-summary-flat.pdf

Life sciences study design

All studies must disclose on these points even when the disclosure is negative.

Sample size	The sample size was estimated to depend on the primary comparison, PreA-only versus No-PreA. The target minimum sample size of 2010 participants (670 participants per study arm) was prespecified based on a power analysis using the preliminary data of 90 patients in the pilot study before study enrollment. This minimum target sample size ensured sufficient power (>80%) for the primary outcome at a significance level of 0.05.
Data exclusions	2,332 patients and their care partners were evaluated for eligibility, with 194 either opting out or being excluded for various reasons (Fig. 2). This left 2,138 patients who were randomly assigned to the PreA-only group (n= 712), PreA-human group (n= 713), or the No PreA group (n= 713) using sealed envelopes for a straightforward 1:1:1 allocation. Of these, 69 patients later chose to opt-out or were removed for different reasons.
Replication	Stratified analyses demonstrated consistent reductions in physician consultation duration. Notably, these reductions were observed across age groups, sex, educational attainment, work status, income levels, medical disciplines (medical medicine, surgery, mix of medical medicine and surgery, pediatrics), study sites (Guilin/Gansu) and participant type (patients/care partners), with PreA-only showing significant reductions compared to No-PreA, and no significant differences compared to PreA-human (Supplementary Figs. 1-4).
Randomization	We used individual-level parallel randomization without stratification, utilizing a computer-generated random sequence for participant assignment to each experimental group.
Blinding	This trial was single-blinded: While the patients knew their group assignments, the physicians were uninformed about the PreA-intervention groups (PreA-only or PreA-human), and the researchers were also unaware of the assignments.

Reporting for specific materials, systems and methods

We require information from authors about some types of materials, experimental systems and methods used in many studies. Here, indicate whether each material, system or method listed is relevant to your study. If you are not sure if a list item applies to your research, read the appropriate section before selecting a response.

Materials & experimental systems

n/a	Involved in the study	
☒	☐	Antibodies
☒	☐	Eukaryotic cell lines
☒	☐	Palaeontology and archaeology
☒	☐	Animals and other organisms
☐	☒	Clinical data
☒	☐	Dual use research of concern
☒	☐	Plants

Methods

n/a	Involved in the study	
☒	☐	ChIP-seq
☒	☐	Flow cytometry
☒	☐	MRI-based neuroimaging

Clinical data

Policy information about clinical studies

All manuscripts should comply with the ICMJE guidelines for publication of clinical research and a completed CONSORT checklist must be included with all submissions.

Clinical trial registration	Chinese Clinical Trial Registry identifier: ChiCTR2400094159
Study protocol	The study protocol is provided in Supplemetary Information.
Data collection	To evaluate the impact of PreA in a real-world setting, we conducted a multicenter, parallel-group RCT across 24 medical disciplines at two tertiary care centers in western China: the First Affiliated Hospital of Guilin Medical University and the Affiliated Hospital of Gansu Medical College, from February 8, 2025, to April 30, 2025.
Outcomes	The primary outcomes were the duration of physician-patient consultations, physician perception of primary-secondary care coordination, and patient perception of ease of communication (assessed using validated questionnaires). Secondary outcomes included: (1) physician workload (measured by number of patients per shift); (2) patient perceived physician attentiveness during visits, patient satisifaction, interpersonal regard, and future acceptability; (3) physician experience with perceived utility of PreA, ease of communication ease with patients, and relief of workload; (4) physician documentation practices (analyzing clinical note content).

Plants

Seed stocks	*Report on the source of all seed stocks or other plant material used. If applicable, state the seed stock centre and catalogue number. If plant specimens were collected from the field, describe the collection location, date and sampling procedures.*
Novel plant genotypes	*Describe the methods by which all novel plant genotypes were produced. This includes those generated by transgenic approaches, gene editing, chemical/radiation-based mutagenesis and hybridization. For transgenic lines, describe the transformation method, the number of independent lines analyzed and the generation upon which experiments were performed. For gene-edited lines, describe the editor used, the endogenous sequence targeted for editing, the targeting guide RNA sequence (if applicable) and how the editor was applied.*
Authentication	*Describe any authentication procedures for each seed stock used or novel genotype generated. Describe any experiments used to assess the effect of a mutation and, where applicable, how potential secondary effects (e.g. second site T-DNA insertions, mosiacism, off-target gene editing) were examined.*
